# Heteromer Nanostars by Spontaneous Self-Assembly

**DOI:** 10.3390/nano7060127

**Published:** 2017-05-31

**Authors:** Caitlin Brocker, Hannah Kim, Daniel Smith, Sutapa Barua

**Affiliations:** 1 Department of Chemical and Biochemical Engineering, Missouri University of Science and Technology, 110 Bertelsmeyer Hall, 1101 N. State Street, Rolla, MO 65409, USA; cebfk6@mst.edu (C.B.); dlsrt8@mst.edu (D.S.); 2 Department of Biological Sciences, Missouri University of Science and Technology, 143 Schrenk Hall, 400 W. 11th St., Rolla, MO 65409, USA; hhk4hf@mst.edu

**Keywords:** nanostar, polylactide-*co*-glycolic acid (PLGA), star shape, tetrakis (hydroxylmethyl) phosphonium chloride (THPC)

## Abstract

Heteromer star-shaped nanoparticles have the potential to carry out therapeutic agents, improve intracellular uptake, and safely release drugs after prolonged periods of residence at the diseased site. A one-step seed mediation process was employed using polylactide-*co*-glycolic acid (PLGA), polyvinyl alcohol (PVA), silver nitrate, and tetrakis(hydroxymethyl)phosphonium chloride (THPC). Mixing these reagents followed by UV irradiation successfully produced heteromer nanostars containing a number of arm chains attached to a single core with a high yield. The release of THPC from heteromer nanostars was tested for its potential use for breast cancer treatment. The nanostars present a unique geometrical design exhibiting a significant intracellular uptake by breast cancer cells but low cytotoxicity that potentiates its efficacy as drug carriers.

## 1. Introduction

Synthesis of self-assembly systems is of significant interest to optimize drug delivery efficacy. Polymeric assembly may offer variation in composition, chemical functionality, size, and shape including disks, rods, spheres, cubes, and filaments to name just a few [1,2,3,4,5,6,7,8,9]. The choice of non-spherical nanoparticles exhibits improved blood circulation time [7], specific receptor binding [10], cellular internalization [9,10], and low phagocytosis compared to its spherical counterparts [11,12]. Non-spherical lipomers composed of poly(methylvinylether-*co*-maleic anhydride) and lipids have shown higher splenic accumulation in rats, rabbits, and dogs and are more effective at evading non-specific uptake by macrophages than spherical lipomers, suggesting a potential for the carrier to be used for drug delivery to the spleen [13]. Star-shaped poly(l-lactide) [14], poly(ethylene-*co*-propylene) [15], and polybutadienes [16] have been synthesized with one end chemically linked to a hydrophilic core, while the other end is functionalized creating a hydrophobic corona [14]. Multistep polymerization reactions have been involved in synthesizing star-shaped particles such as dendrimers and micelles [8,17,18,19,20]. These macromolecules require polymers of various lengths and particle generations. The interest in such systems originates from, in addition to their biocompatible properties, their applications in slow release drug delivery systems, bioresorbable surgical sutures, and surgical implants. To this end, heteromer star-shaped nanoparticles are synthesized with independent sets of arm chains attached to a single polymer core. Poly(lactide)-*co*-glycolic acid (PLGA) polymer is chosen for its biodegradable characteristics by hydrolytic cleavage of ester groups in the physiological microenvironment, forming non-toxic (biodegradable and biocompatible) lactic acid and glycolic acid groups [21]. While PLGA scaffold has been widely used for developing drug delivery systems [21,22,23,24,25,26,27,28,29,30], the preparation of heteromer-shaped PLGA particles is unknown. Herein, a combinatorial method is described where a phosphonium salt—tetrakis (hydroxymethyl) phosphonium chloride (THPC)—is adsorbed on PLGA in the presence of silver nitrate seed. THPC has been used as a crosslinker of hydrogels [31] and a reducing agent for metal nanoparticles [32,33]. It is hypothesized that the chemical reduction of silver in the presence of THPC impregnated in PLGA nanoparticles causes the intraparticle morphology to vary. In addition, due to its chemical structure, THPC is available in aqueous solution and cytocompatible for pharmaceutical applications. The focus of this study is the synthesis of heteromer star-shaped nanoparticles as an anticancer drug delivery platform, combining a self-assembled THPC reducing agent with PLGA and the resulting nanostars for intracellular uptake by breast cancer cells.

## 2. Materials and Methods

### 2.1. Synthesis of PLGA-THPC Heteromer Nanostars

Most reagents were purchased from Sigma-Aldrich (St. Louis, MO, USA) unless specified otherwise. THPC was kindly donated by Kattesh Katti from the University of Missouri Columbia (Columbia, MO, USA). Nanostars were developed using a silver core surrounded by THPC particles coated with PLGA polymer [34]. Polyvinyl alcohol (PVA; MW 33,000–70,000) of 5 mg was dissolved in 10 mL of reverse osmosis water at 80 °C. Once this solution was cooled to room temperature, 1 mL of 1 mg/mL THPC was pipetted into the PVA solution. Fifty milligrams of PLGA was dissolved in 2 mL of acetone and added dropwise to the PVA solution under sonication. Prepared PLGA particles were mixed with 450 μL of 0.1 N silver nitrate (AgNO_3_) solution (Acros Organics, Fisher Scientific, Waltham, MA, USA). The mixture was gently mixed, transferred into round, shallow Petri dishes with a thickness of ~3 mm, and exposed to 8 W of UV light (254 nm) for 40 min. In a separate beaker, trisodium citrate (TSC; Alfa Aesar, Haver Hill, MA, USA) of 0.7 mmol was added to a mixture of 10 mg/mL PVA. The silver-nucleated PLGA solution was transferred into the TSC-PVA mixture and stirred for 5 min. Finally, 100 μL of ascorbic acid was added to the mixture and stirred vigorously at room temperature for 5 min. PVA was removed by repeated washing using water and centrifugation.

### 2.2. Measurement of THPC in Nanostars

A standard curve was generated by plotting absorbance values versus various concentrations of THPC for measuring THPC concentrations in nanostars. Given the chemical structure of THPC, the chloride ion on THPC is highly reactive with the silver ion in silver nitrate (${\left({\mathrm{HOCH}}_{2}\right)}_{4}\mathrm{PCl}+{\mathrm{AgNO}}_{3}\to \mathrm{AgCl}↓$). This reaction was utilized to generate the THPC standard curve for measuring its soluble concentrations. Briefly, equal parts of THPC and silver nitrate (${\mathrm{AgNO}}_{3}$) were mixed in a 96-well plate (Corning, Corning, NY, USA) at different concentrations. The formation of silver chloride (AgCl) precipitates was detected by measuring the absorbance at 395 nm using a microplate reader (BioTek Synergy 2, BioTek, Winooski, VT, USA). Water was used as a blank.

### 2.3. Characterization of Nanostars

The morphology and size of PLGA-THPC nanostars were examined using a transmission electron microscope (TEM; Tecnai F20; FEI company, Hillsboro, OR, USA) at an accelerating voltage of 120 kV. A drop of 10 μL of a previously prepared PLGA-THPC nanostar suspension was pipetted onto carbon-coated copper grids (Ted Pella, Redding, CA, USA) and air-dried. The diameter of nanostars was measured using ImageJ (version 1.45S, NIH, Bethesda, MD, USA) for at least 20 particles. The size and surface charges of nanostars were measured by dynamic light scattering using a Nanoseries Zetasizer ZS 90 (Malvern Instruments Ltd., Malvern, Worcestershire, UK), with backscattering detection at 90°.

### 2.4. Quantification of THPC Release

The PLGA-THPC nanostars were suspended in water at both pH 7.4 and 6.2. Each sample of 5 mL was placed in a 37 °C water bath to mimic the body temperature. At *t* = 0, 0.5, 2, 4, 8, 24, 36, and 72 h, a 500 μL solution was withdrawn from each sample. The solution was analyzed for THPC concentration using the THPC standard curve. Phosphate buffer saline (PBS) could not be used in this study, as it has been used in other drug release studies [9,35,36], due to the interference of sodium chloride with silver chloride during the absorbance measurement assay.

### 2.5. Intracellular Uptake

MDA-MB-231 breast cancer cell monolayers were seeded at a density of 50,000 cells/mL in 8-well chamber plates and grown overnight. The medium was replaced with fresh medium including nanostars for 2 h of incubation with THPC alone as a control. The nuclei were stained by 4′,6-diamidino-2-phenylindole (DAPI). The cells were washed using PBS. The intracellular accumulation of nanostars was visualized using a fluorescence microscope (Zeiss, Oberkochen, Germany) equipped with a transmitted light illuminator (Zeiss, Oberkochen, Germany), fluorescence filter set of 390/450 ex/em, 63× water immersion objective, an Axiocam camera (Zeiss, Oberkochen, Germany), and ZEN2 Pro software (Zeiss, Oberkochen, Germany).

### 2.6. In Vitro Cytotoxicity of Heteromer Nanostars in MDA-MB-231 Breast Cancer Cells

MDA-MB-231 cells (ATCC, Manassas, VA, USA) were cultured in RPMI 1640 medium supplemented with 10% FBS (Corning) and 1% (100 units/mL) penicillin-streptomycin (Gibco, Gaithersberg, MD, USA) in a 5% CO_2_ and 37 °C incubator. The cells were plated in 96-well tissue plates (Corning) at a density of 10,000 cells/well in 200 μL of medium. PLGA-THPC nanostars were added to the cells at 0, 0.1, 0.5, 1, 5, 10, 15, 20, and 25 μg/mL of THPC. PBS was used as a negative control (100% live cells) along with Triton X-100 as a positive control (100% dead cells). After 3 h of incubation, the medium was replaced; cells were incubated for another 72 h. Live cells were measured using the live/dead assay (Life Technologies, Carlsbad, CA, USA). Briefly, the medium was removed followed by an addition of 2 μM calcein in PBS to stain the live cells in each well. Cells were incubated for 30 min at room temperature. The fluorescence intensity of calcein AM was measured at an excitation/emission of 485/528 using a plate reader (BioTek Synergy 2, BioTek, Winooski, VT, USA). The percent inhibition of cell growth was calculated using the following equation:

$$\%\;inhibition\;of\;cell\;growth=\frac{F.I._{PBS\;treated\;cells}-F.I._{samples}}{F.I._{PBS\;treated\;cells}}\times 100.$$

### 2.7. Statistical Analysis

Each experiment was carried out with three independent experiments of at least triplicate measurements. The mean differences and standard deviations were evaluated.

## 3. Results and Discussion

### 3.1. Synthesis of Heteromer Nanostars

**Figure 1:**
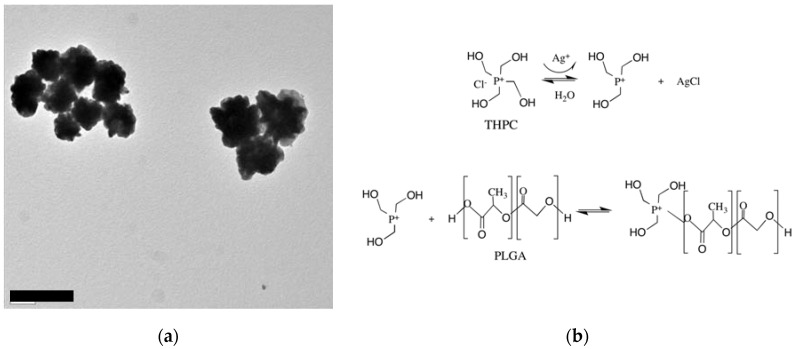
(**a**) TEM image of heteromer PLGA-THPC star-shaped nanoparticles (scale bar = 500 nm); (**b**) A hypothesized mechanism of PLGA-THPC nanostar assembly.

**Figure 2:**
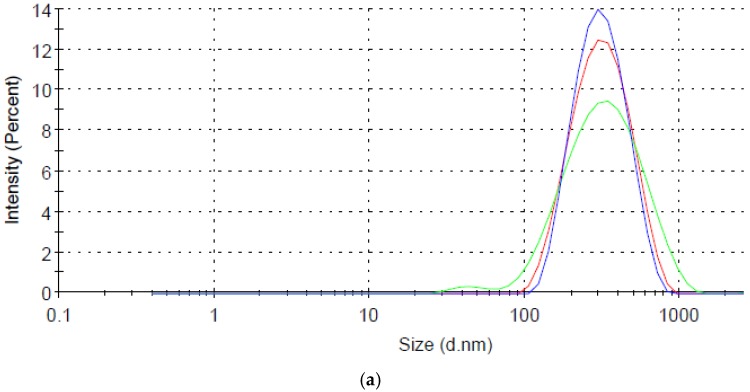

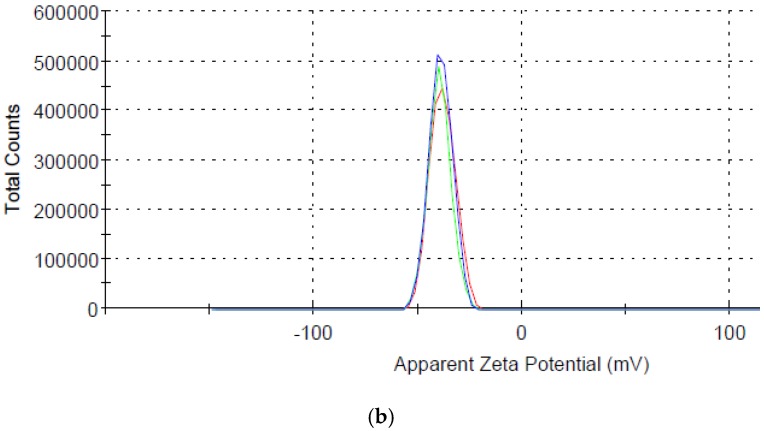
(**a**) Size distribution of PLGA-THPC nanostars; (**b**) surface charges by dynamic light scattering in water (pH 6.8). The three colored (blue, red, and green) curves represent the reproducibility in nanostar size and surface charges, as synthesized from three separate batches.

**Figure 3:**
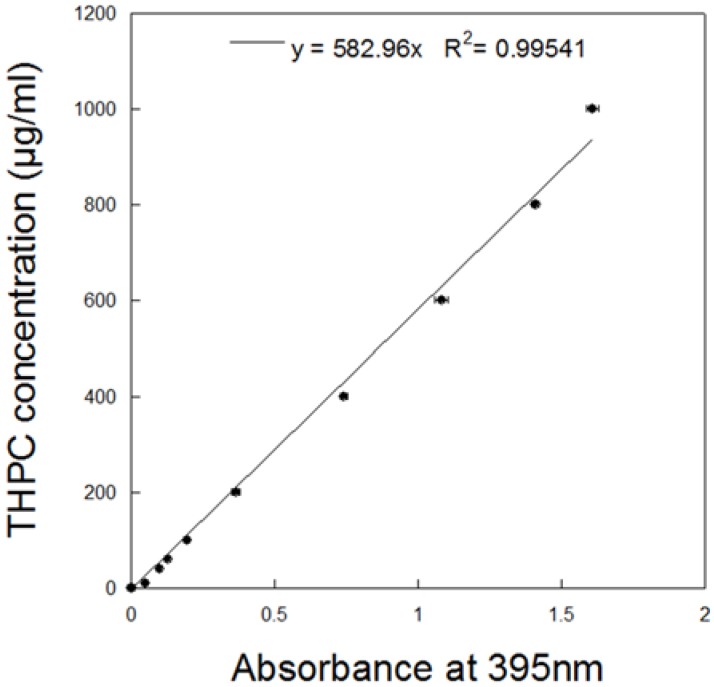
Standard curve demonstrating the linear relationship between absorbance of reacted silver chloride at 395 nm and THPC concentration in μg/mL.

A star-like growth is a seed-mediated process in the presence of the PLGA core, THPC molecules, and a silver ion [34]. A silver-polymer composite star synthesis protocol was employed to encapsulate THPC in PLGA nanoparticles (Figure 1a). Three steps are required for this synthesis: phase separation, PLGA-THPC nanostar synthesis, and photoreduction. PLGA-THPC nanostars were formed employing oil in a water emulsion technique, also known as phase separation. By dissolving PLGA polymers in acetone to create an oil phase and gently pipetting the oil phase dropwise to the water phase under sonication, PLGA-THPC nanoparticles were formed. When the nanoparticles were placed in a Petri dish and irradiated with a UV light, THPC served as a reducing agent for Ag^+^ in solution, as shown mechanistically in Figure 1b. THPC is a strong reducing agent for synthesizing nanoparticles [32,37]. Photoreduction, along with adding a strong reducing agent, by reducing silver nitrate, allowed for a change in its morphology to a star shape. The TEM image describes the formation of a uniform shape of PLGA–Ag nanoparticles and star-shaped morphology of a THPC-encapsulated PLGA silver nanoparticle. The average number of arms (sharp corners) was calculated at 10 ± 2. The average hydrodynamic radius was ~315 ± 140 nm (Figure 2a) that reflects their average ensemble size when dispersed in PBS. The three intensity distribution curves (blue, red, and green) in Figure 2a represent light scattering data of nanoparticles from three different batches. All samples showed the highest peak at around 315 nm in size, indicating the reproducibility of the presented nanostar synthesis method. The differences in percent intensity on the *y*-axis are due to variation in particle concentrations. A small peak at around 40 nm is seen in the green curve and may be due to impurities in the system or PLGA fragments from biodegradation in the storing solution. However, the strong peak at 315 nm shows that the majority of particles are in this range. The surface charge of the nanoparticles was −39 ± 5.5 mV (Figure 2b) in water (pH 6.8), indicating a stable nanoparticle suspension in the aqueous medium. The encapsulation of THPC was investigated from silver chloride precipitation and absorbance curve. A calibration curve was constructed for THPC by measuring absorbance at 395 nm versus different concentrations of THPC (Figure 3). A linear curve (y=mx) was generated to find out the slope and regression coefficient (*R*^2^ > 0.99) using the least squares regression method. The percentage encapsulation efficiency was calculated ~60% ± 5% based on the initial loading of the THPC.

### 3.2. THPC Release

**Figure 4:**
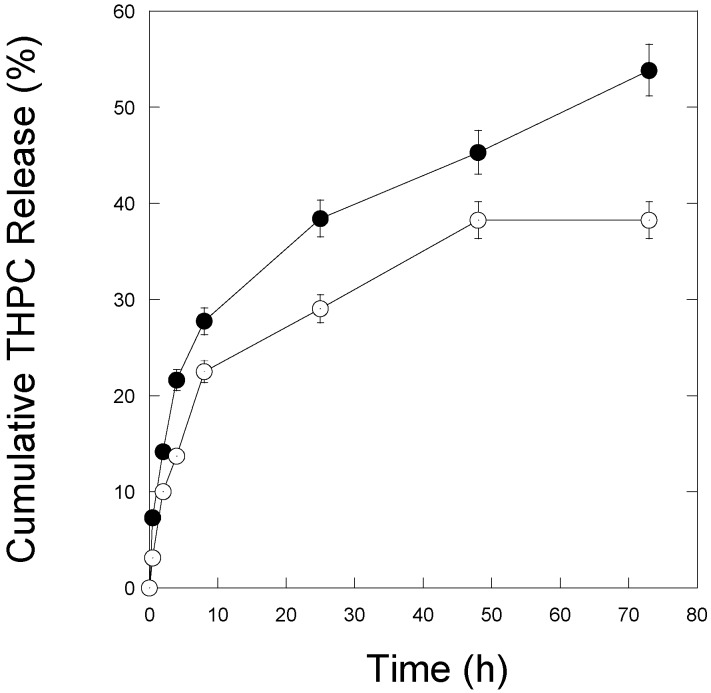
The cumulative release amount of THPC from PLGA-THPC nanostars at pH 6.2 (solid filled circles) and pH 7.4 (open circles) as determined by the silver chloride precipitation and absorbance (A_395_) assay. The complete release profile assay was conducted in a 37 °C water bath. The release rate is faster in breast cancer cells mimicking pH 6.2 than the body pH at 7.4.

The percentage of THPC release at different time intervals and different pH values are shown in Figure 4. A similar amount (~5%) of THPC was released from PLGA-THPC nanostars up to 5 min in PBS buffer at both pH 6.2 (solid point) and pH 7.4 (open point). The incorporated THPC release occurred due to the hydrolysis of PLGA in PBS through cleavage of ester linkages in its backbone [21]. The biodegradation is faster in the slightly acidic medium. Faster and higher release of THPC was observed when nanostars were exposed to a pH 6.2 environment compared to a pH 7.4 environment. At pH 7.4 (open point), it took nearly 72 h to reach ~40% release, while ~75% of the release rate was reached at pH 6.2 (solid point). It is known that the pH of an inflammatory disease site such as a tumor and a cytoplasm is lower than that of blood plasma [38]. The THPC release data shows a higher release at lower pH than the physiological blood pH.

### 3.3. Cellular Uptake and Cytotoxicity in Breast Cancer Cells

**Figure 5:**
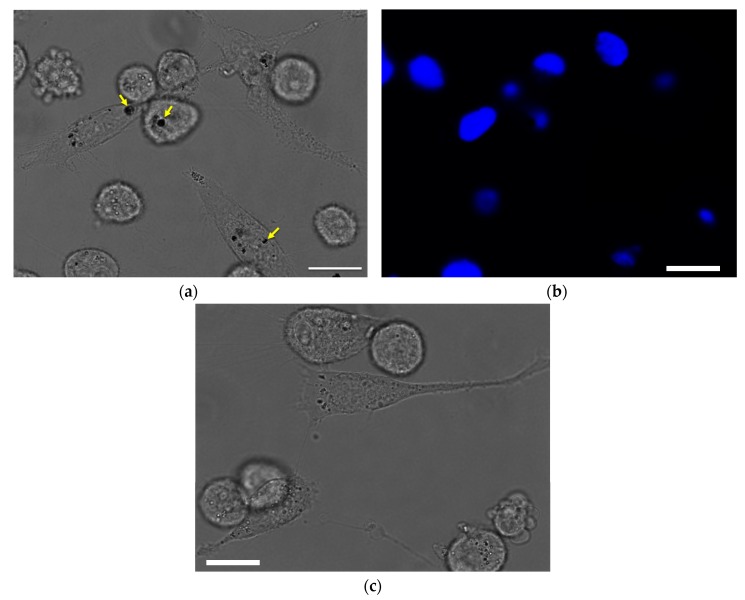
(**a**) Phase contrast image showing the intracellular uptake of PLGA-THPC nanostars by MDA-MB-231 cells. The black aggregates (as indicated by yellow arrows) indicate the spatial distribution of the nanostars; (**b**) Fluorescent nuclei of MDA-MB-231 breast cancer cells after 72 h incubation with nanostars; (**c**) Control MDA-MB-231 breast cancer cells were incubated with similar amount of THPC solution without any nanoparticles. No black spots in this control confirm the intracellular uptake of nanostars in (**a**). Scale bar = 20 μm.

**Figure 6:**
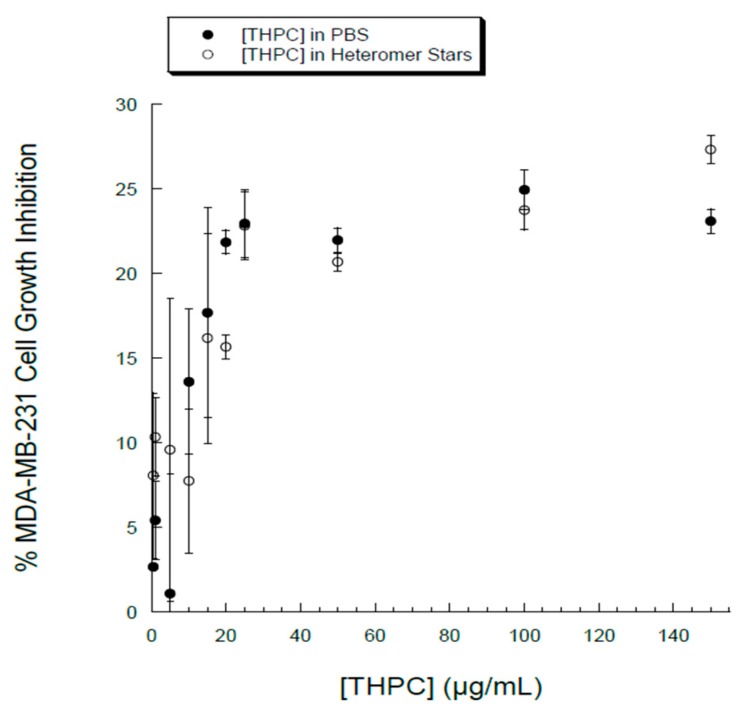
Cytotoxicity of PLGA-THPC nanostars (filled circles) as measured by its dose-dependent effects on MDA-MB-231 breast cancer cell growth inhibition. THPC solution (open circles) was used as a control.

The therapeutic effects of drug nanoparticles depend on their internalization and sustained retention inside diseased cells [20]. In order to evaluate the intracellular localization and in vitro cytotoxicity of THPC, breast cancer cell MDA-MB-231 was chosen. Phase microscopy was used to visualize the internalization and distribution of PLGA-THPC nanostars in MDA-MB-231 cells (Figure 5). The nanostars accumulated inside endosomes after adsorptive endocytosis through fluid phase pinocytosis and clathrin-coated endocytosis process [39,40]. PLGA nanoparticles have been shown to degrade in acidic endolysosomal compartments [40,41], where it may release THPC in a sustained manner. The phosphonium cation of THPC facilitates the accumulation from endosomes into the mitochondria, which is promising for mitochondria-targeted cancer therapy [42,43]. The nanostars accumulated inside the endosomes after intracellular internalization. The cytotoxic activity of PLGA-THPC star-shaped nanoparticles was evaluated in MDA-MB-231 cells (Figure 6; filled circles) at varying concentrations. The *x*-axis represents the concentrations of THPC in the PLGA-THPC star-shaped nanoparticles. The corresponding effects by THPC solution alone are shown in Figure 6 (open circles). The PLGA-THPC nanostars did not show much cytotoxicity against MDA-MB-231 cells, with up to 25% MDA-MB-231 cell death at 25 μg/mL of THPC, which followed a trend similar to a solution of THPC alone. Surprisingly, THPC nanostars yielded a conspicuously higher cell growth inhibition than did pure THPC, without showing statistically significant differences. Although the underlying mechanism of such behavior is unknown, a possible explanation could be related to the enhancement in intracellular uptake and the reduction in drug efflux by P-glycoprotein (P-gp) membrane transporter proteins [44,45,46] for the nanostar-mediated THPC delivery. The drug efflux pump, P-gp, is overexpressed in MDA-MB-231 breast cancer cells [47]. Nanoparticles have been developed to increase the intracellular concentration of drugs in cancer cells by circumventing the P-gp exerted resistance [46,48]. The mechanisms of carriers to overcome P-gp have been reported for various drug delivery systems, including PLGA [49], *N*-(2-hydroxypropyl)methacrylamide (HPMA) drug conjugates [50], polymeric micelles [51,52], hybrid lipid nanoparticles [53], lipid-based nanoparticles [54], and liposomes [55]. Doxorubicin-loaded PLGA/lipid nanoassemblies increased drug uptake and enhanced cytotoxicity in MCF-7 human breast cancer cells bypassing drug resistance [56]. Doxorubicin-loaded nanoparticles increased intracellular drug concentration and efficiently suppressed P-gp expression in multidrug resistant osteosarcoma cell lines [57]. Nanoparticles of doxorubicin and curcumin overcome multidrug resistance in multiple in vivo models, including multiple myeloma, acute leukemia, and prostate and ovarian cancers [58]. The low cytotoxicity effects by nanostars indicate a new possibility of using this geometrical shape as a potential drug carrier for cancer treatment.

## 4. Conclusions

Heteromer nanostars containing ten arm species with PLGA and THPC were prepared. Simple mixing of biodegradable polymers and subsequent linking reaction with THPC and silver ions successfully yielded heteromer nanostars. The particles are highly stable as predicted from their high zeta potential value. Approximately 75% THPC was released in pH mimicking buffer conditions. In particular, breast cancer cells took up the nanostars inside the endosomes, where pH was slightly more acidic (pH 6) compared to the blood pH at 7.4 [39,40]. Furthermore, the nanostars were evaluated to induce cytotoxicity in breast cancer cells. Although the cytotoxic effect by the nanostars alone is low, the features of heteromer chains were clearly demonstrated in terms of their potential drug delivery carriers in breast cancer treatment.
